# Monitoring Over Time of Pathological Complete Response to Neoadjuvant Chemotherapy in Breast Cancer Patients Through an Ensemble Vision Transformers‐Based Model

**DOI:** 10.1002/cam4.70482

**Published:** 2024-12-18

**Authors:** Maria Colomba Comes, Annarita Fanizzi, Samantha Bove, Luca Boldrini, Agnese Latorre, Deniz Can Guven, Serena Iacovelli, Tiziana Talienti, Alessandro Rizzo, Francesco Alfredo Zito, Raffaella Massafra

**Affiliations:** ^1^ Laboratorio di Biostatistica e Bioinformatica I.R.C.C.S. Istituto Tumori “Giovanni Paolo II” Bari Italy; ^2^ Unità Operativa Complessa di Radioterapia Oncologica Fondazione Policlinico Universitario Agostino Gemelli I.R.C.C.S Rome Italy; ^3^ Unità Operativa Complessa di Oncologia Medica I.R.C.C.S. Istituto Tumori “Giovanni Paolo II”Bari Bari Italy; ^4^ Department of Medical Oncology Hacettepe University, Cancer Institute Ankara Turkey; ^5^ Trial Office, I.R.C.C.S. Istituto Tumori “Giovanni Paolo II” Bari Bari Italy; ^6^ Struttura Semplice Dipartimentale di Oncologia Medica per la Presa in Carico Globale del Paziente Oncologico “Don Tonino Bello” I.R.C.C.S. Istituto Tumori “Giovanni Paolo II” Bari Italy; ^7^ Unità Operativa Complessa di Anatomia Patologica, I.R.C.C.S. Istituto Tumori “Giovanni Paolo II” Bari Italy

**Keywords:** breast cancer, ensemble model, Neoadjuvant chemotherapy, pathological complete response, vision transformers

## Abstract

**Background:**

Morphological and vascular characteristics of breast cancer can change during neoadjuvant chemotherapy (NAC). Dynamic contrast‐enhanced magnetic resonance imaging (DCE‐MRI)‐acquired pre‐ and mid‐treatment quantitatively capture information about tumor heterogeneity as potential earlier indicators of pathological complete response (pCR) to NAC in breast cancer.

**Aims:**

This study aimed to develop an ensemble deep learning‐based model, exploiting a Vision Transformer (ViT) architecture, which merges features automatically extracted from five segmented slices of both pre‐ and mid‐treatment exams containing the maximum tumor area, to predict and monitor pCR to NAC.

**Materials and Methods:**

Imaging data analyzed in this study referred to a cohort of 86 breast cancer patients, randomly split into training and test sets at a ratio of 8:2, who underwent NAC and for which information regarding the pCR status was available (37.2% of patients achieved pCR). We further validated our model using a subset of 20 patients selected from the publicly available I‐SPY2 trial dataset (independent test).

**Results:**

The performances of the proposed model were assessed using standard evaluation metrics, and promising results were achieved: area under the curve (AUC) value of 91.4%, accuracy value of 82.4%, a specificity value of 80.0%, a sensitivity value of 85.7%, precision value of 75.0%, F‐score value of 80.0%, and G‐mean value of 82.8%. The results obtained from the independent test show an AUC of 81.3%, an accuracy of 80.0%, a specificity value of 76.9%, a sensitivity of 85.0%, a precision of 66.7%, an F‐score of 75.0%, and a G‐mean of 81.2%.

**Discussion:**

As far as we know, our research is the first proposal using ViTs on DCE‐MRI exams to monitor pCR over time during NAC.

**Conclusion:**

Finally, the changes in DCE‐MRI at pre‐ and mid‐treatment could affect the accuracy of pCR prediction to NAC.

## 1 Introduction

Neoadjuvant chemotherapy (NAC) in breast cancer is becoming a standard treatment due to its advantages over adjuvant therapy, such as assessing response during treatment, improving surgical outcomes, and enabling surgical de‐escalation without compromising efficacy [1]. Additionally, NAC helps identify patients with a better prognosis by assessing pathological complete response (pCR), a crucial predictor of favorable outcomes. pCR, determined through surgery after therapy as the absence of cancer cells in the breast and/or axillary lymph nodes, is essential for tailoring subsequent treatment strategies and improving patient prognosis [2, 3, 4]. Achieving pCR can significantly impact the planning of treatments, including recalibrating procedures before and after surgery [5, 6, 7]. Moreover, recent findings have highlighted the critical role of integrating early pCR predictions into the contemporary breast cancer management framework, especially regarding the minimization of mastectomies when pCR is achieved [8]. However, pCR is evaluated from surgical specimens, making early prediction and monitoring during NAC challenging.

Different histologic patterns of residual disease also play a fundamental role in the prognosis of breast cancer patients treated with NAC [9]. Various studies have proposed methods to assess pCR early, including mammography, ultrasonography, and dynamic contrast‐enhanced magnetic resonance imaging (DCE‐MRI). Among these, DCE‐MRI has been found to be most reliable [10]. Clinical protocols suggest acquiring DCE‐MRI scans at multiple time points during NAC, namely, before treatment (MRI T1), during treatment (MRI T2), and at the end of treatment, to monitor breast cancer progression. Radiological information from these scans can potentially indicate early pCR.

Radiologists manually identify tumor areas in MRI exams and assess changes in tumor diameter over time using Response Evaluation Criteria in Solid Tumors (RECIST) [11]. However, this method is operator‐dependent and prone to errors. Radiomics, which converts bioimages into quantitative data, has emerged as a more efficient method for predicting therapy efficacy [12, 13]. Conventional radiomics involves extracting handcrafted features, which are still operator‐dependent.

To address these limitations, deep learning‐radiomic workflows have been developed to automatically extract features from raw scans without human intervention. Convolutional neural networks (CNNs) have shown promise in medical image analysis compared to handcrafted methods [14, 15]. Recent studies have used CNNs for pCR prediction using MRI during NAC, that is, to classify patients into either pCR class or non‐pCR class [16, 17, 18, 19].

Vision Transformers (ViTs), a recent development in deep learning, split images into patches and use self‐attention mechanisms to capture global dependencies between image tokens [20, 21]. To our knowledge, there is limited research on using ViTs for monitoring pCR during NAC in breast cancer. Our study employs a ViT‐based ensemble model on MRI T1 and T2 scans from breast cancer patients undergoing NAC at our Institute. The model was tested on a dataset extracted from patients enrolled at our Institute, as well as on a subset of the publicly available I‐SPY2 trial database. This database is a comprehensive collection of clinical and imaging data from breast cancer patients undergoing neoadjuvant therapy as part of the adaptive phase II I‐SPY2 trial [22, 23, 24].

## 2 Materials and Methods

### 2.1 Segmentation Algorithm

This retrospective study was approved by the Scientific Board of the Istituto Tumori “Giovanni Paolo II” in Bari, Italy‐Prot. 1168/CE. The DCE‐MRI scans referred to the patients enrolled at our Institute are contrast‐enhanced T1‐weighted axial exams counting from 160 to 360 slices. Each slice was acquired six times: a single pre‐contrast image and five post‐contrast images corresponding to approximately each minute after injection of gadobutrolo (Gadovist, Bayer, Germany), at a dose of 0.1 mmol/kg of body weight and flow rate of 1.5–2 mL/s, followed by 20 mL of saline solution, were acquired in the prone position with a dedicated seven‐channel breast coil on a 1.5 Tesla Philips scanner (Achieva, Philips Medical Systems, Best, the Netherlands).

In the case of the public I‐SPY2 trial database, the slices were acquired both prior to the injection and at six subsequent time points afterward, with the MRI scans performed at either 3 T or 1.5 T. We selected the independent test set from the I‐SPY2 trial dataset to ensure that the images corresponding to these patients exhibited homogenous characteristics compared to those of the patients from our institution. A key criterion for patient selection was that the MRIs were scanned at 1.5 T.

We automatically segmented and then extracted quantitative imaging information from second post‐contrast DCE‐MRI examinations acquired at pre‐ and mid‐treatment because the contrast during the early post‐injection phase between tumor and the surrounding tissue is optimal to a finer analysis of morphological characteristics, as demonstrated in the current state of the art [25]. We further segmented the pre‐ and mid‐treatment DCE‐MRI exams for the independent test specifically analyzing the images acquired at the second time point following contrast injection.

The segmentation algorithm we implemented refers to an extension of the one proposed by Wei et al. [26], combined with the application of some morphological operators. It was applied along all the slices composing the two MRI exams under study.

Here, we briefly explain the procedure performed on one slice. First, the chest wall (CW) region of interest (ROI) was detected. After masking the CW ROI, an image containing both breasts was obtained (see panel A of Figure S1). Two images showing the two breasts separately were generated and the corresponding mean gray intensity was computed to identify the breast containing the tumor mass (BROI, see panel B of Figure S1). Finally, by applying some morphological operators, a sequence of five slices related to the BROI comprising the maximum tumor area were extracted (BROI slices, see panel C of Figure S1). Before given in input to the learning model, the extracted slices were resized using zero‐padding as it preserves both the shape and size of the tumor, which is crucial for maintaining the accuracy of tumor volume, that is, a significant predictor of pCR status as emphasized in previous studies [27, 28]. Thus, by using zero padding, we ensured that the original resolution of the tumor was preserved, while the surrounding empty space in the bounding box was padded. This approach prevented any alterations to the tumor's original structure or distortions that could have occurred with interpolation, thereby minimizing any potential negative impact on model performance.

For more details, please refer to Data S1.

### 2.2 Learning Model

**FIGURE 1:**
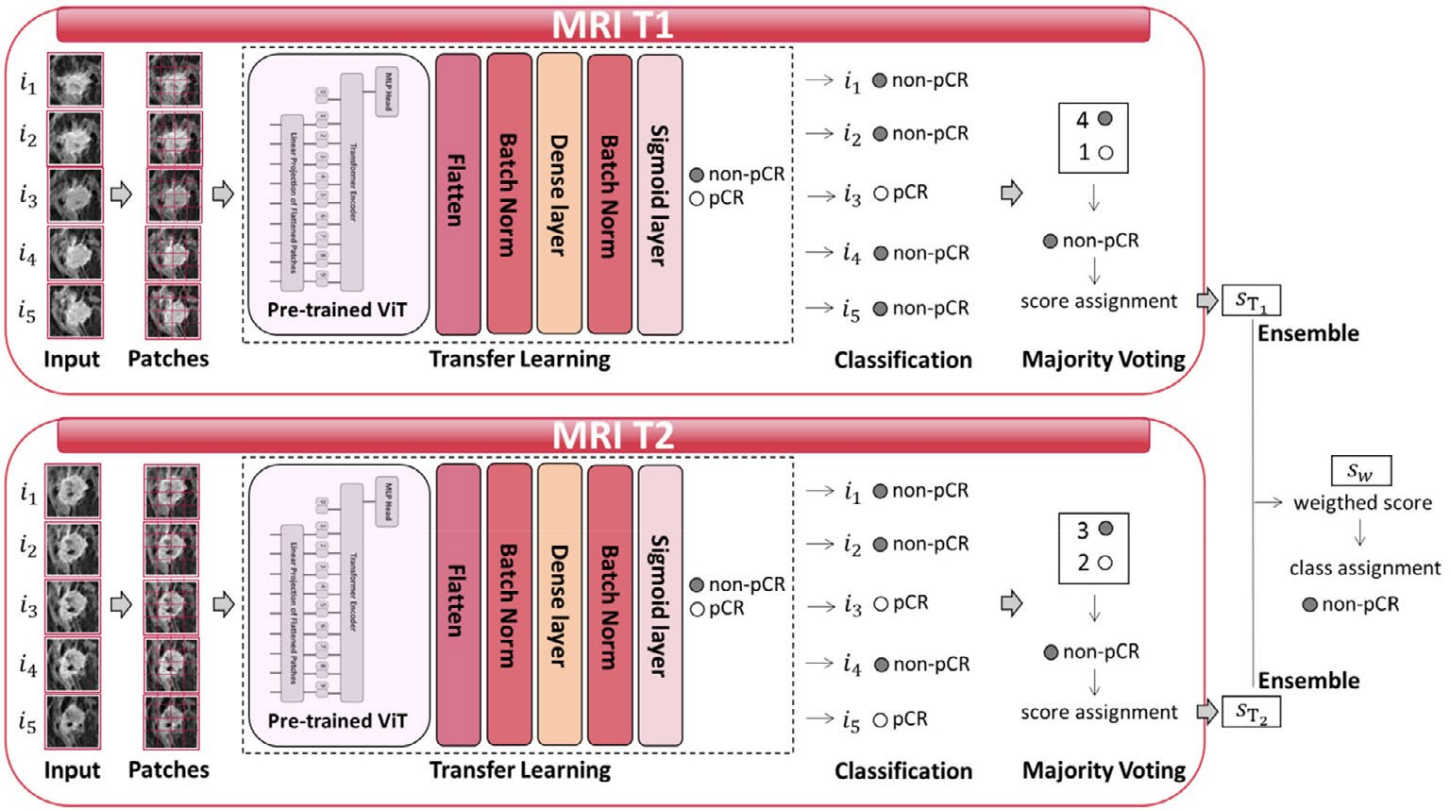
Workflow of the proposed learning method. Ensemble model composed by two baseline models sharing the same backbone architecture, but separately analyzing five BROI slices related to MRI T1 and MRI T2, respectively, comprised the ensemble model. The backbone architecture of the two models mainly consisted of two modules: The transfer learning module based on a ViT architecture and the majority voting module.

An ensemble deep learning‐based predictive model to monitor pCR over time during NAC was designed. We formulated a binary classification task to predict and distinguish patients who reached or did not reach pCR at the end of therapy (pCR class and non‐pCR class, respectively). Two baseline models sharing the same backbone architecture, but separately analyzing the five BROI slices related to MRI T1 and MRI T2, respectively, comprised the ensemble model (Figure 1). The backbone architecture of the two models mainly consisted of two modules: a transfer learning module based on ViTs and a majority voting module.

#### 2.2.1 Transfer Learning Module

Transfer learning consists of leveraging features learned on one task by pre‐trained neural networks to be applied to a new task, that, in our case, is pCR prediction. The most common workflow of transfer learning envisages freezing layers from a previously trained model containing information learned during a previous training phase and then adding and training some trainable layers on top of the frozen layers to turn the old features into predictions on the dataset under analysis [29].

We decided to use transfer learning rather than designing a customized ad‐hoc network because of the relatively small size of the dataset at disposal. On this kind of datasets, research works of the state of the art have demonstrated the successful application of transfer learning techniques with promising and also generalizable results on independent validation cohorts [30]. The pre‐trained network used to build our transfer learning model was a ViT architecture (ViTb_16).

The BROI slices were reshaped to 224 × 224 size images and partitioned into 16 patches per image, in order to be given as input to the pre‐trained ViT architecture. Among the possible ViT networks, we decided to use a ViT architecture employing patches of size 16 × 16 as input because of its robustness against performance degradation and computational complexity [20]. Within the pre‐trained architecture, the obtained patches are flattened and mapped through a trainable linear projection to produce a series of embedded image patches. To perform the classification task, an encoder receives the sequence of the embedded picture patches, together with positional data, which add positioning information to the input, and a learnable class embedding sequence whose values represent the corresponding classification outcome. The output of the transformer encoder is sent to a multilayer perceptron (MLP) head to return the classification. To apply transfer learning, the last layer of the network was replaced with some stacking trainable layers, which are a flattening layer, a batch normalization layer, a dense layer with Gaussian Error Linear Unit (GELU) activation function together with an L2 regularizer, another batch normalization layer, and a final dense layer as a classifier with a sigmoid activation function. The model was trained and the data split into training and test sets according to an 8:2 ratio. Both sets contained the same proportion between the two classes. All the BROI slices associated to one patient were part of either the training set or the test set depending on whether the patient was assigned to the training set or the test set, respectively. Finally, the transfer learning module returned a classification score for each of the five BROI slices related to each patient. We further validated the developed learning model using an independent test set, which comprised a subset of 20 patients from the I‐SPY2 trial database, selected based on the criteria summarized in the first sub‐sections of Methods and Results. In this instance, the entire cohort of 86 patients from our institute was utilized as the training set.

#### 2.2.2 Majority Voting Module

A majority voting technique was performed (see Figure 1) to obtain a unique classification score per patient. The final class assignment at the end of a model corresponds to the class that was most frequently assigned for the five BROI slices. The corresponding classification score was computed as the maximum/minimum score of the models labeling the patient into the pCR/non‐pCR class, if the class assigned by the majority voting was the pCR/non‐pCR class, respectively.

The responses obtained for each patient by the two models, separately analyzing the five BROI slices related to MRI T1 and MRI T2, were joined together, thus resulting in an ensemble model. Let $s_{T_{1}}$ and $s_{T_{2}}$ be the classification scores returned for a patient belonging to the test set by models exploiting MRI T1 and MRI T2, respectively. Then, a weighted score, $s_{w}$, was obtained by implementing the ensemble procedure consisting of weighting each of the two scores $s_{T_{i}}$ $\left(i=1,2\right)$ with a term expressing the ability of the respective model to discern pCR and non‐pCR classes on the training set, namely, the $x\left({\mathrm{AUC}}_{{\mathrm{MRI}}_{T_{i}}}\right)$. To assure the $s_{w}$ value lying in the range [0;1], a normalizer term was also multiplied:

(1) normalizer=1AUCMRIT1+AUCMRIT2

(2) $$\begin{array}{c} s_{w}=s_{T_{1}}\times \left({\mathrm{AUC}}_{{\mathrm{MRI}}_{T_{1}}}\times \text{normalizer}\right)\\ +s_{T_{2}}\times \left({\mathrm{AUC}}_{{\mathrm{MRI}}_{T_{2}}}\times \text{normalizer}\right) \end{array}$$

### 2.3 Competing Pre‐Trained Architectures

To assess the robustness of the proposed learning model based on ViT, the transfer learning modules of the two baseline models were replaced using some pre‐trained CNN architectures, known as good performing in the field of computer vision applied to biomedicine. They are ResNet101 [31], Densenet201 [32], and Xception [33]. ResNet101 architecture is a 101 layer‐net belonging to the class of residual CNNs, which make use of stacking residual blocks to train much deeper networks with the aim of maintaining compelling performances. It receives 224 × 224 size images as input. The DenseNet201 model is composed of layers receiving additional inputs from all preceding layers and passing their feature‐maps to all subsequent layers. It receives 224 × 224 size images as input. Xception is a 71‐layer deep architecture, whose function is to apply the filters on each of the depth map and then compress the input space using 1 × 1 convolution across the depth. It receives 299 × 299 size images as input. To obtain a fair comparison with the proposed learning model, the classification layer of these networks was replaced by the same trainable layers used for the pre‐trained ViT architecture, except for the activation function of the dense layer, which in this case was Rectified Linear Unit (ReLu).

### 2.4 Implementation Details

All the trainable models were trained for 30 epochs using a batch size of 8. To address the class imbalance issue (37.2% of pCR cases), focal loss rather than binary cross entropy error was defined as the loss function of the networks [34]. The Adam optimization algorithm was used to optimize the weights of the network [35] with a starting learning rate of 10^−4^. To prevent overfitting, data augmentation based on random flip horizontally and vertically, random rotation with angles in the range [−20, 20] degrees with a step of 5°, and randomly contrast adjustment with a factor of 0.2, was implemented in the training phase. The implementation code was written and run using ColabPro Notebook.

### 2.5 Explainability: LIME Algorithm

The predictions obtained at the transfer learning level were visually interpreted employing the local interpretable model‐agnostic explanations (LIME) [36, 37]. Basically, the algorithm generates a new dataset of “perturbed” samples with the corresponding predictions of the network. On this dataset, an interpretable model, which is weighted by the proximity of the sampled instances to the instance for which we want to have an explanation, is trained. The learned model should be a good approximation of the predictions locally. In the case of explanation of image samples, variations of the images are generated by segmenting them into “superpixels” and turning superpixels off or on. A heatmap over the raw images highlights the most important superpixels, that is, those regions mainly contributing in the decision‐making process. With respect to the label predicted by the network (pCR/non‐pCR), the regions which positively contribute to the assignment of that image into the predicted class are colored green, while the negatively contributing superpixels are colored red. LIME picks the a priori defined threshold value to select the number of top contributing superpixels. In this case, we set the threshold equal to 20.

### 2.6 Performance Evaluation

The performance of all the introduced models in assigning patients belonging to the test set to the either pCR class or non‐pCR class was evaluated in terms of area under the curve (AUC) as well as standard metrics, which are accuracy, sensitivity, specificity, and precision. Two other metrics, namely, F1‐score and geometric mean (G‐mean), which have been evaluated as suitable to assess an appropriate performance measure for imbalanced datasets [38], were also computed. While F1‐score evaluates the relative contribution of precision and sensitivity as equal, G‐mean takes into account the balance between classification performances on both classes, thus avoiding overfitting of the most numerous class as well as underfitting of the class with the minor number of subjects. Finally, the bootstrap paired t‐test was utilized to evaluate the AUC values of the proposed model in comparison to competing models, ensuring a robust statistical analysis that accommodates the limitations associated with the dataset size [39]. A result was considered statistically significant when the p‐value returned was less than 0.05.

## 3 Results

### 3.1 Data Collection

A cohort of 86 breast cancer patients who underwent NAC at the same institute from 2017 to 2022 was enrolled. The following criteria were required for inclusion: (i) primary breast cancer confirmed using core needle biopsy before the beginning of therapy; (ii) no metastasis ab initio; (iii) availability of both pretreatment and mid‐treatment breast DCE‐MRI scans (MRI T1 and MRI T2, respectively), where mid‐treatment MRI were acquired after three or four cycles of chemotherapy; (iv) absence of any treatment before NAC; (v) availability of information regarding pCR achievement. Among the patients included in this study, 32 (37.2%) achieved pCR at completion of the entire course of NAC (pCR class), while 54 (62.8%) have not reached pCR (non‐pCR class), as shown in Table 1. Other clinical information is summarized in the same table. The variables ER (Clone EP1 DAKO) and PgR (Clone PgR636) were reported as negative if ER and PgR were equal to 0; positive if ER and PgR assumed values higher than or equal to 1%, respectively, whereas Ki67 (Clone MIB1 DAKO) was reported in percentage. The HER2 (polyclonal Rabbit Anti‐Human c‐erb 2 Oncoprotein) variable was performed according to the ASCO‐CAP guidelines; the grading values were assessed in agreement with Elston Classification.

**TABLE 1:** Clinical characteristics referred to the patients enrolled at our institute. Absolute values and percentages are reported (percentages in round brackets). For age and Ki67, the median value and first (q_1_) and third (q_3_) quartiles of the distribution are indicated in squared brackets.

	pCR class	non‐pCR class
Overall (abs.; %)	32 (37.2%)	54 (62.8%)
Age (years)		
Median; [q_1_, q_2_]	50 [43.5, 61.0]	47 [41.0, 62.0]
Grading		
G1 (abs.; %)	1 (3.1%)	2 (3.75)
G2 (abs.; %)	1 (3.1%)	16 (29.6%)
G3 (abs.; %)	28 (87.5%)	33 (61.1%)
NA (abs.; %)	2 (6.3%)	3 (5.6%)
ER		
Negative (abs.; %)	16 (50.0%)	14 (25.9%)
Positive (abs.; %)	16 (50.0%)	40 (74.1%)
PgR		
Negative (abs.; %)	25 (78.1%)	23 (42.6%)
Positive (abs.; %)	7 (21.9%)	31 (57.4%)
Ki67 (%)		
Median; [q_1_, q_3_]	55 [35.0, 75.0]	30 [20.0, 40.0]
HER2		
Negative (abs.; %)	13 (40.6%)	36 (66.7%)
Positive (abs.; %)	19 (59.4%)	18 (33.3%)

The I‐SPY2 trial dataset includes several clinical variables such as hormone receptor (HR) status, HER2 status, age, menopausal status, and race. We selected an independent test set of 20 patients from the I‐SPY2 trial to ensure that the proportion of pCR and non‐pCR classes was consistent with that of our own database. Table 2 summarizes the main clinical characteristics related to the independent test.

**TABLE 2:** Clinical characteristics referred to the independent test. Absolute values and percentages are reported (percentages in round brackets). For age, the median value and first (q_1_) and third (q_3_) quartiles of the distribution are indicated in squared brackets.

	pCR class	non‐pCR class
Overall (abs.; %)	7 (35.0%)	13 (65.0%)
Age (years)		
Median; [q_1_, q_2_]	47 [40.5, 56.5]	51 [39.0, 52.3]
HR		
Negative (abs.; %)	5 (71.4%)	4 (30.8%)
Positive (abs.; %)	2 (28.6%)	9 (69.2%)
HER2		
Negative (abs.; %)	4 (57.1%)	9 (69.2%)
Positive (abs.; %)	3 (42.9%)	4 (30.8%)

### 3.2 Performance Evaluation

**FIGURE 2:**
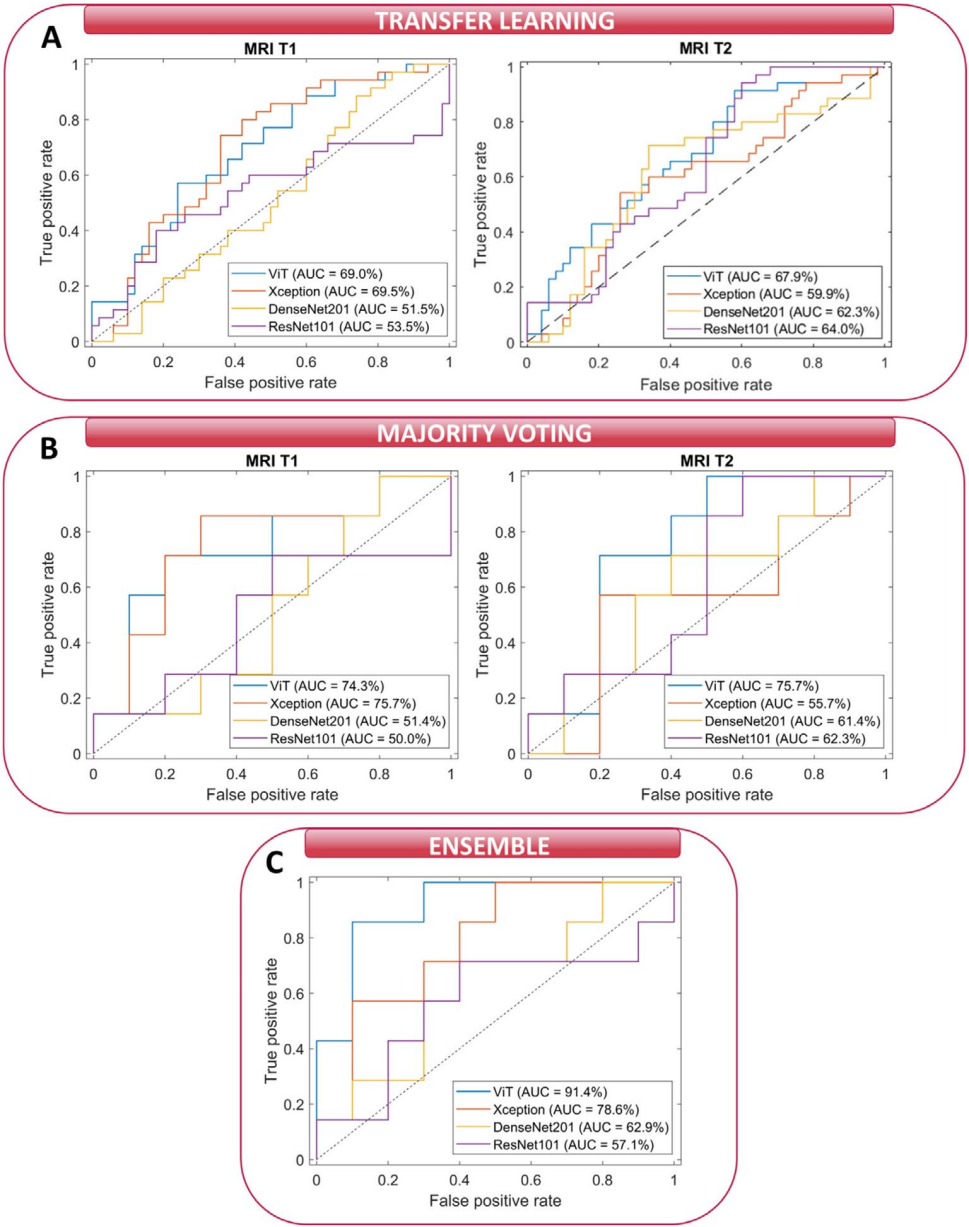
Comparison of ROC curves and the corresponding AUC values for the test set related to our institute's cohort. (A) ROC curves of the two baseline models at the transfer learning level when varying the transfer learning module composing the baseline models, either ViT or CNN architectures, (B) ROC curves of the two baseline models at the majority voting level when varying the transfer learning module composing the baseline models, either ViT or CNN architectures, and (C) ROC curves at the ensemble level when varying the transfer learning module composing the baseline models, either ViT or CNN architectures.

Receiver operating characteristic (ROC) curves for both baseline models exploiting MRI T1 and MRI T2 (panels A and B) and for the ensemble model (panel C) are depicted in Figure 2. The corresponding AUC values are also reported. Specifically, the curves and AUC values were computed at the transfer learning level (panel A), at the majority voting level (panel B), and at the ensemble level (panel C) by varying the transfer learning module embedded in the baseline models (either ViT or CNNs). From looking at panels A and B, a dual comparison, that is, between the two baseline models and among diverse embedded transfer learning modules, could be performed: ViT and Xception architectures reached the best AUC values for the MRI T1 model; ViT and Densenet201 modules outperformed the other modules for the MRI T2 model. Anyway, the ViT architecture achieved the most stable AUC values between the two models (69.0% and 74.3% for the MRI T1 model and 67.9% and 75.7% for the MRI T2 model at the transfer learning level and majority voting level, respectively).

**FIGURE 3:**
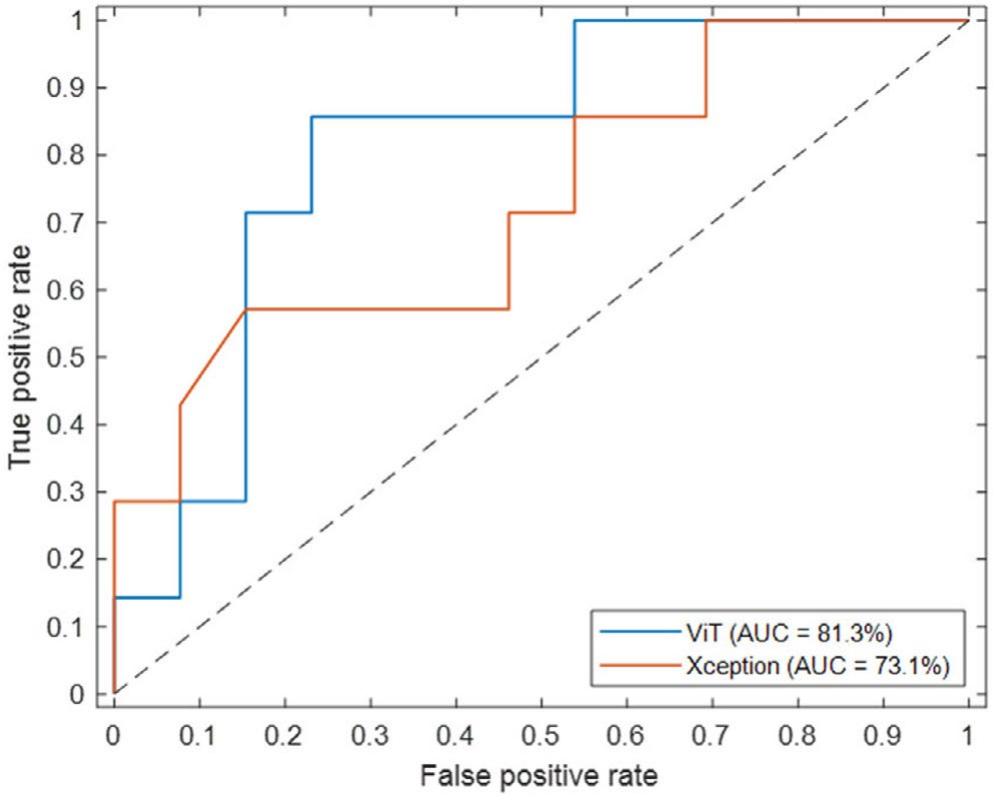
Comparison of ROC curves and the AUC values obtained from the ViT‐based ensemble model and the best competing model, Xception, in the independent test set.

In contrast to the AUC values obtained at transfer learning and majority voting level, differences among AUC values obtained by the ensemble model based on ViT (91.4%) and the ensemble models based on CNNs modules were more evident. Among the CNNs, the best AUC value was reached in correspondence of Xception architecture (78.9%). We found a significant p‐value in the comparison of the AUC values returned by our model with the competing models (ViT versus Xception: *p* = 0.0045, ViT versus DenseNet201: *p* = 10^−4^, ViT versus ResNet101: *p* = 10^−5^), suggesting that there is a statistically significant difference in their performance in discriminating between classes. Overall, the ViT‐based ensemble model obtained the best performances in terms of other standard metrics, as summarized in Table 3: accuracy value of 82.4%, sensitivity value of 85.7%, precision value of 75.0%, F‐score value of 80.0%, and G‐mean value of 82.8%. Specificity is the only metric with a lower value than Xception, but more balanced with respect to sensitivity (80.0% vs. 90.0%). We tested the ViT‐based ensemble model and the best competing model, that is, Xception, over the independent test reaching an AUC value of 81.3% and 73.1%, an accuracy value of 80.0% and 70.0%, a specificity value of 76.9% and 77.0%, a sensitivity value of 85.0% and 57.1%, a precision value of 66.7% and 57.1%, a F‐score value of 75.0% and 57.1%, and a G‐mean value of 81.2% and 66.3%, respectively. Figure 3 shows the ROC curves related to the two abovementioned models. In this instance, our ViT‐based model demonstrated superior performance compared to the competing model, with a significant p‐value of 0.01 indicating a difference between the two AUC values.

**TABLE 3:** Summary of evaluation metrics for the ensemble model when varying the transfer learning module composing the baseline models, either ViT or CNN architectures. For each metric, bold values indicate the optimal achieved values.

	Ensemble model			
	ViT	Xception	Densenet201	ResNet101
AUC (%)	**91.4**	78.6	62.9	57.1
Accuracy (%)	**82.4**	70.6	64.7	64.7
Specificity (%)	80.0	**90.0**	57.1	60.0
Sensitivity (%)	**85.7**	42.9	70.0	71.4
Precision (%)	**75.0**	**75.0**	57.1	55.6
F‐score (%)	**80.0**	54.6	57.1	62.5
G‐mean (%)	**82.8**	62.1	63.3	65.5

### 3.3 Explainability Results

**FIGURE 4:**
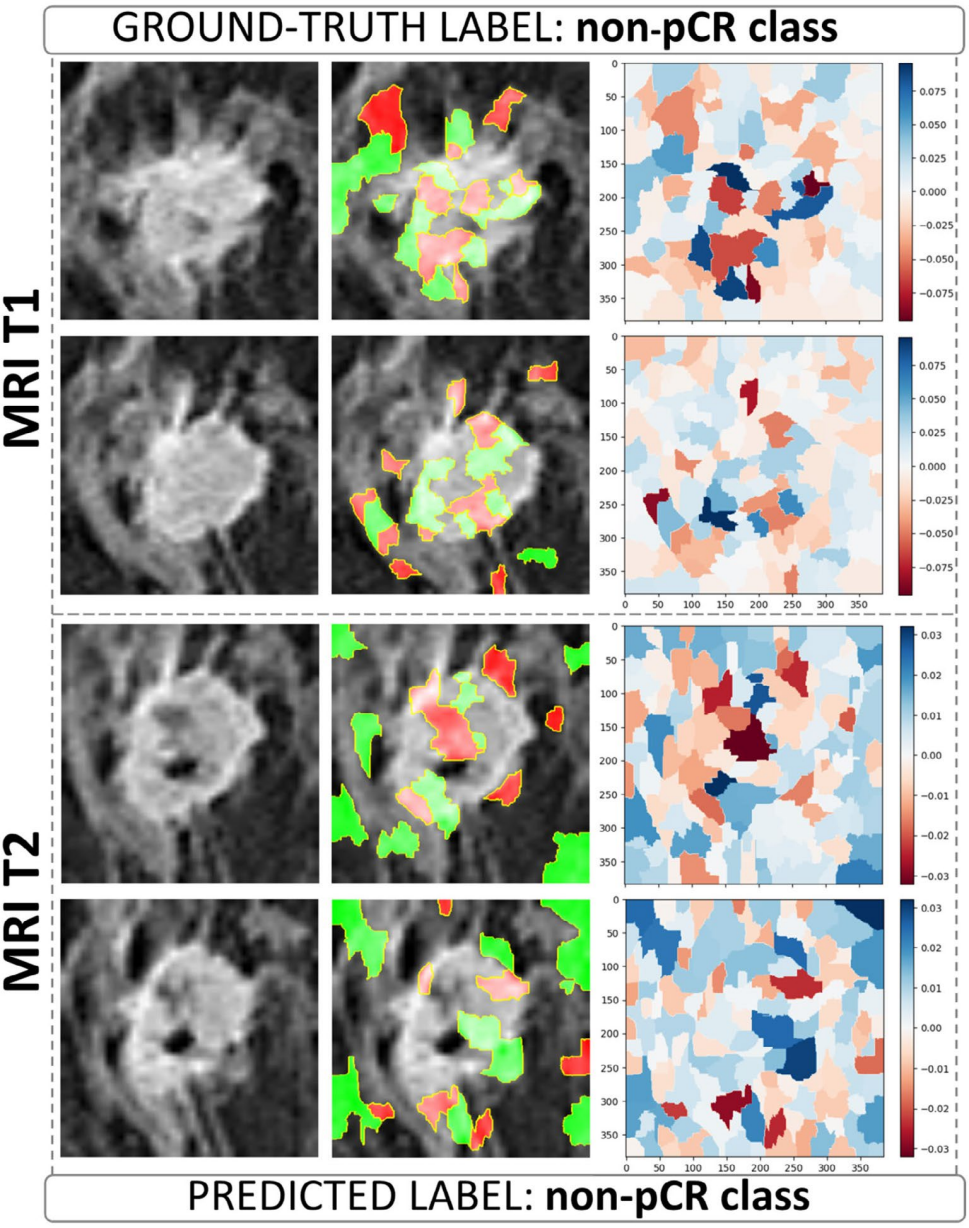
Visualization of LIME superpixels in positive and negative regions applied to two BROI slices from a non‐pCR patient in our institute's cohort, correctly classified by both the MRI T1 and MRI T2 models utilizing a transfer learning module based on the ViT architecture. The panels on the left show the raw slices. The central ones depict the raw slices overlaid by the most contributing superpixels, where the red color highlights the negatively contributing superpixels to the assignment to non‐pCR class, whereas the green represents otherwise. The panels on the right represent heatmaps where color intensity is a measure of importance of all the superpixels generated on the raw slices (blue for a positive contribution and red for a negative contribution).

**FIGURE 5:**
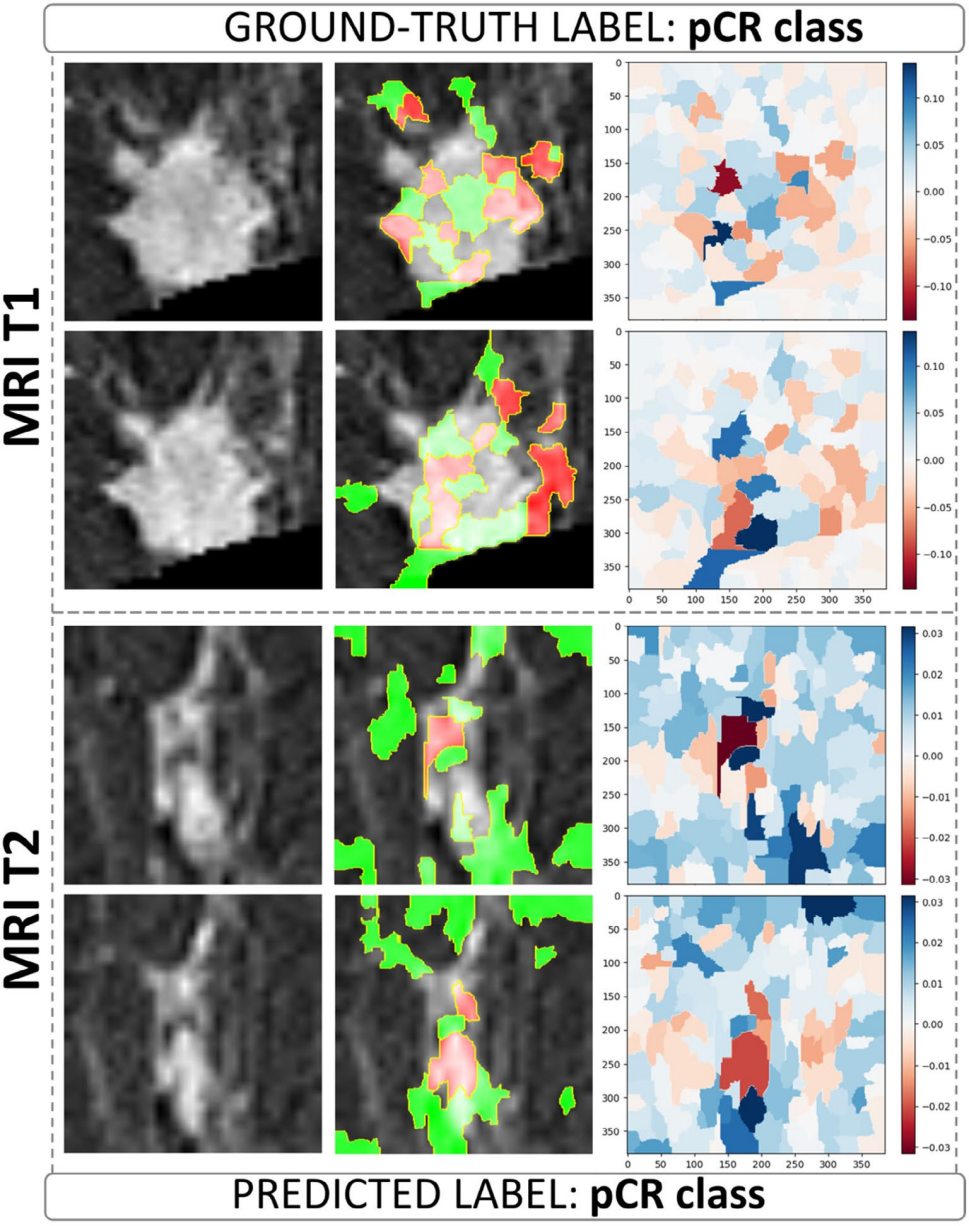
Visualization of LIME superpixels in positive and negative regions applied to two BROI slices from a pCR patient in our institute's cohort, correctly classified by both the MRI T1 and MRI T2 models utilizing a transfer learning module based on the ViT architecture. The left panels show the raw slices. The central panels depict the raw slices overlaid by the most contributing superpixels, where the red color highlights the negatively contributing superpixels to the assignment to pCR class, whereas the green represents otherwise. The right panels represent heatmaps where color intensity is a measure of importance of all the superpixels generated on the raw slices (blue for a positive contribution and red for a negative contribution).

The visual explanation of the decision‐making process underlying the ViT architecture at the transfer learning level was obtained by applying the LIME algorithm. The visualization of LIME superpixels in positive and negative regions applied to two BROI slices related to a non‐pCR/pCR patient correctly classified by both MRI T1 and MRI T2 models are depicted in Figures 3, 4 and 5, respectively, representing images from patients at our institute. Meanwhile, the images from patients in the independent test set can be found in Figures S2 and S3. The raw slices are shown alongside the raw slices overlaid by the most contributing superpixels, where the red color highlights the negatively contributing superpixels to the assignment to non‐pCR/pCR class, whereas the green represents otherwise.

The panels on the right present heatmaps where color intensity represents the importance of each superpixel generated from the raw slices, with blue indicating a positive contribution and red a negative contribution to the assigned class. In these heatmaps, a higher color intensity reflects a greater significance of the corresponding superpixel. As shown, the most important superpixels, whether contributing positively or negatively, are primarily concentrated within the intratumoral area and along the lesion's edges. This suggests that both the internal features of the tumor and the boundaries of the lesion play a crucial role in determining the classification, highlighting their relevance in the model's decision‐making process.

However, other valuable superpixels refer to the surrounding peritumoral area, that is, the site of peripheral neo angiogenesis. Examples of visualization of LIME superpixel positive and negative regions applied to two BROI slices related to a non‐pCR/pCR patient misclassified by both MRI T1 and MRI T2 models with transfer learning module as ViT architecture are reported in Figures S4 and S5.

## 4 Discussion

We used pre‐ and mid‐treatment DCE‐MRI exams to evaluate quantitative information of tumor heterogeneity during NAC to predict and monitor pCR achievement in breast cancer patients.

An ensemble model that integrates multi‐period image features was developed. The focus of our work is part of an active research field, whose objective is the early prediction of pCR by analyzing image data acquired before or during NAC to address the clinical need of improving and personalizing treatment planning, with the aim of sparing patients from potentially ineffective and/or toxic treatment.

Most of the models developed in the field employed either conventional radiomics [40, 41, 42] or CNNs [16, 17, 18, 19, 43, 44]. However, the exploitation of the recently introduced ViT architectures for this application area is not widespread yet. As far as we know, Tong et al. [45] were pioneers in applying a ViT‐based approach on image data to predict pCR in breast cancer. They developed a multi‐time‐point ViT, taking in input the ultrasound (US) exams acquired before and after NAC, in order to predict pCR shortly before surgery.

In our study, we used a ViT architecture on DCE‐MRI scans acquired before and in the middle of the treatment. We did not analyze the scan at the end of NAC because our main goal is to provide clinicians a support to evaluate possible changes in the ongoing NAC treatment.

This study is the first building block to achieve this ambitious purpose. The encouraging results achieved on both the test set related to our institute's cohort and the independent test set are competitive when compared to the state of the art. Among the more recent studies analyzing pre‐ and mid‐treatment MRI data to predict pCR to NAC in breast cancer patients, Eun et al. [40] carried out a texture analysis on mid‐treatment axial MRI exam achieving an AUC value equal to 82%.

Fun et al. [46] defined predictive models merging radiomics features from pre‐ and mid‐ treatment MRI scans also integrated with molecular subtype information, finally obtaining an AUC value of 80.9%. Li et al. [18] combined conventional radiomics features with deep learning features extracted by a cutting‐edge CNN from pre‐ and mid‐treatment MRI exams, achieving an AUC value of 90.0%.

This study has some limitations. The results from our model's performance present an interesting contrast, particularly when examining the AUC values achieved on different datasets. On the test set derived from our patients, the model attained an AUC of 91.4%. This high value indicates that our model is highly effective at distinguishing between the classes within our specific cohort, likely due to the relevance of the features utilized and the model's ability to capture the underlying patterns present in this dataset.

However, when evaluated on an independent test set extracted from a public dataset, the performance decreased to an AUC of 84%. While this remains a commendable performance, the difference between the two AUC values prompts important considerations. The reduction in performance may suggest that the model's effectiveness is somewhat context‐dependent, highlighting the potential variability in results when applied to different populations or datasets. Moreover, the public dataset may encompass variations in clinical settings or imaging techniques that were not fully represented in our internal cohort.

This underscores the importance of validating models across diverse datasets to ensure their robustness and generalizability. Additionally, it highlights the necessity for further investigation to comprehend the factors that contribute to the observed differences in outcomes. In this context, exploring image harmonization techniques could be beneficial. Approaches such as neural style transfer, histogram matching, and domain adaptation methods can help align images from different sources, mitigating the impact of variability in imaging conditions. By implementing these innovative harmonization techniques, we could potentially enhance model performance across varying datasets and improve its applicability in diverse clinical settings.

However, it is essential to recognize that the current analysis should be considered in a hypothesis‐generating study aimed at identifying initial trends and associations, rather than leading to definitive conclusions. While our findings provide valuable insights, further research is required to validate these hypotheses and ultimately enhance clinical decision‐making. We recognize the need for further validation on larger and more diverse datasets to establish the robustness and generalizability of our findings across various clinical contexts. Moreover, as part of future work, we will consider incorporating end‐of‐treatment scans, which could provide a meaningful benchmark for assessing the predictive performance of our model for early pCR prediction. This approach would allow for a more comprehensive evaluation of the effectiveness of the proposed model and its clinical applicability in monitoring treatment responses.

In conclusion, we developed an ensemble ViT‐based model to extract quantitative data from pre‐ and mid‐treatment DCE‐MRI, predicting pCR achievement. With further validation, it could guide early treatment decisions and personalization. Additionally, localization maps offer visual insights into the model's decision‐making, enhancing clinician understanding and trust.

## Author Contributions

**Maria Colomba Comes:** conceptualization (equal), formal analysis (equal), investigation (equal), methodology (equal), software (equal), visualization (equal), writing – original draft (equal), writing – review and editing (equal). **Annarita Fanizzi:** conceptualization (equal), formal analysis (equal), methodology (equal), validation (equal), writing – original draft (equal), writing – review and editing (equal). **Samantha Bove:** validation (equal), writing – original draft (equal), writing – review and editing (equal). **Luca Boldrini:** writing – review and editing (equal). **Agnese Latorre:** conceptualization (equal), investigation (equal), validation (equal), writing – original draft (equal), writing – review and editing (equal). **Deniz Can Guven:** writing – review and editing (equal). **Serena Iacovelli:** writing – review and editing (equal). **Tiziana Talienti:** writing – review and editing (equal). **Alessandro Rizzo:** validation (equal), writing – review and editing (equal). **Francesco Alfredo Zito:** validation (equal), writing – review and editing (equal). **Raffaella Massafra:** conceptualization (equal), funding acquisition (equal), project administration (equal), supervision (equal), validation (equal), writing – original draft (equal), writing – review and editing (equal).

## Ethics Statement

The study was conducted according to the guidelines of the Declaration of Helsinki and approved by the Scientific Board of Istituto Tumori “Giovanni Paolo II”, Bari, Italy‐ prot 1168/CE. The authors affiliated to Istituto Tumori “Giovanni Paolo II”, IRCCS, Bari, are responsible for the views expressed in this article, which do not necessarily represent the ones of the Institute.

## Consent

“Informed consent” for publication was collected for all the patients involved in the study, except for patients who are dead or not reachable, as it is a retrospective sudy (Garante della Privacy n. 9/2016 in data 15 dicembre 2016).

## Conflicts of Interest

The authors declare no conflicts of interest.

## Supporting information


**Figure S1.** Segmentation algorithm. (A) Chest wall (CW) ROI identification, (B) breast ROI (BROI) identification to choose which breast contains tumor, and (C) BROI slices identification.
**Data S1**. Segmentation algorithm.

**Figure S2.** Visualization of LIME superpixels in positive and negative regions applied to two BROI slices from a non‐pCR patient in the independent test, correctly classified by both the MRI T1 and MRI T2 models utilizing a transfer learning module based on the ViT architecture. The panels on the left show the raw slices. The central ones depict the raw slices overlaid by the most contributing superpixels, where the red color highlights the negatively contributing superpixels to the assignment to non‐pCR class, whereas the green represents otherwise. The panels on the right represent heatmaps where color intensity is a measure of importance of all the superpixels generated on the raw slices (blue for a positive contribution, and red for a negative contribution).

**Figure S3.** Visualization of LIME superpixels in positive and negative regions applied to two BROI slices from a pCR patient in the independent test, correctly classified by both the MRI T1 and MRI T2 models utilizing a transfer learning module based on the ViT architecture. The panels on the left show the raw slices. The central ones depict the raw slices overlaid by the most contributing superpixels, where the red color highlights the negatively contributing superpixels to the assignment to non‐pCR class, whereas the green represents otherwise. The panels on the right represent heatmaps where color intensity is a measure of importance of all the superpixels generated on the raw slices (blue for a positive contribution, and red for a negative contribution).

**Figure S4.** The visualization of LIME superpixels in positive and negative regions applied to two BROI slices related to a non‐pCR patient misclassified by both MRI T1 and MRI T2 models with a transfer learning module as ViT architecture. The left panels show the raw slices. The central panels depict the raw slices overlaid by the most contributing superpixels, where the red color highlights the negatively contributing superpixels to the assignment to non‐pCR class, whereas the green represents otherwise. The right panels represent heatmaps where color intensity is a measure of importance of all the superpixels generated on the raw slices (blue for a positive contribution, and red for a negative contribution).

**Figure S5.** The visualization of LIME superpixels in positive and negative regions applied to two BROI slices related to a pCR patient misclassified by both MRI T1 and MRI T2 models with a transfer learning module as ViT architecture. The left panels show the raw slices. The central panels depict the raw slices overlaid by the most contributing superpixels, where the red color highlights the negatively contributing superpixels to the assignment to non‐pCR class, whereas the green represents otherwise. The right panels represent heatmaps where color intensity is a measure of importance of all the superpixels generated on the raw slices (blue for a positive contribution, and red for a negative contribution).

## Data Availability

Data from this study are available upon request since data contain potentially sensitive information. The data request may be sent to the scientific direction (e‐mail: dirscientifica@oncologico.bari.it).
