# Supplementary figures and images for: Monitoring Over Time of Pathological Complete Response to Neoadjuvant Chemotherapy in Breast Cancer Patients Through an Ensemble Vision Transformers‐Based Model
Source: Cancer Med. 2024 Dec 18;13(24):e70482. doi: 10.1002/cam4.70482 (PMC11653217; doi:10.1002/cam4.70482)

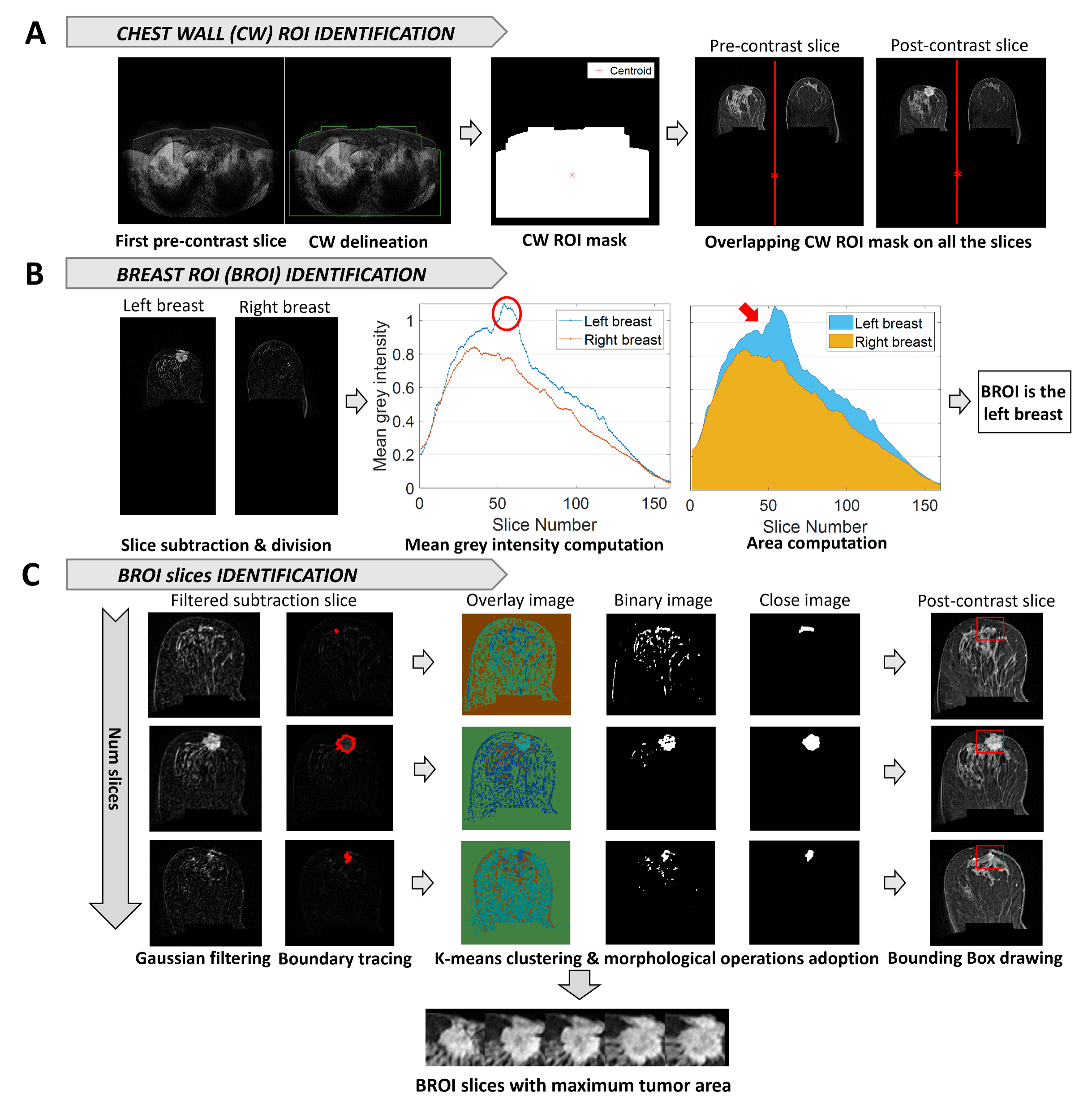

Supplement: Supplementary file 1 — Figure S1. Segmentation algorithm. (A) Chest wall (CW) ROI identification, (B) breast ROI (BROI) identification to choose which breast contains tumor, and (C) BROI slices identification. Data S1. Segmentation algorithm. [file CAM4-13-e70482-s002.tif]

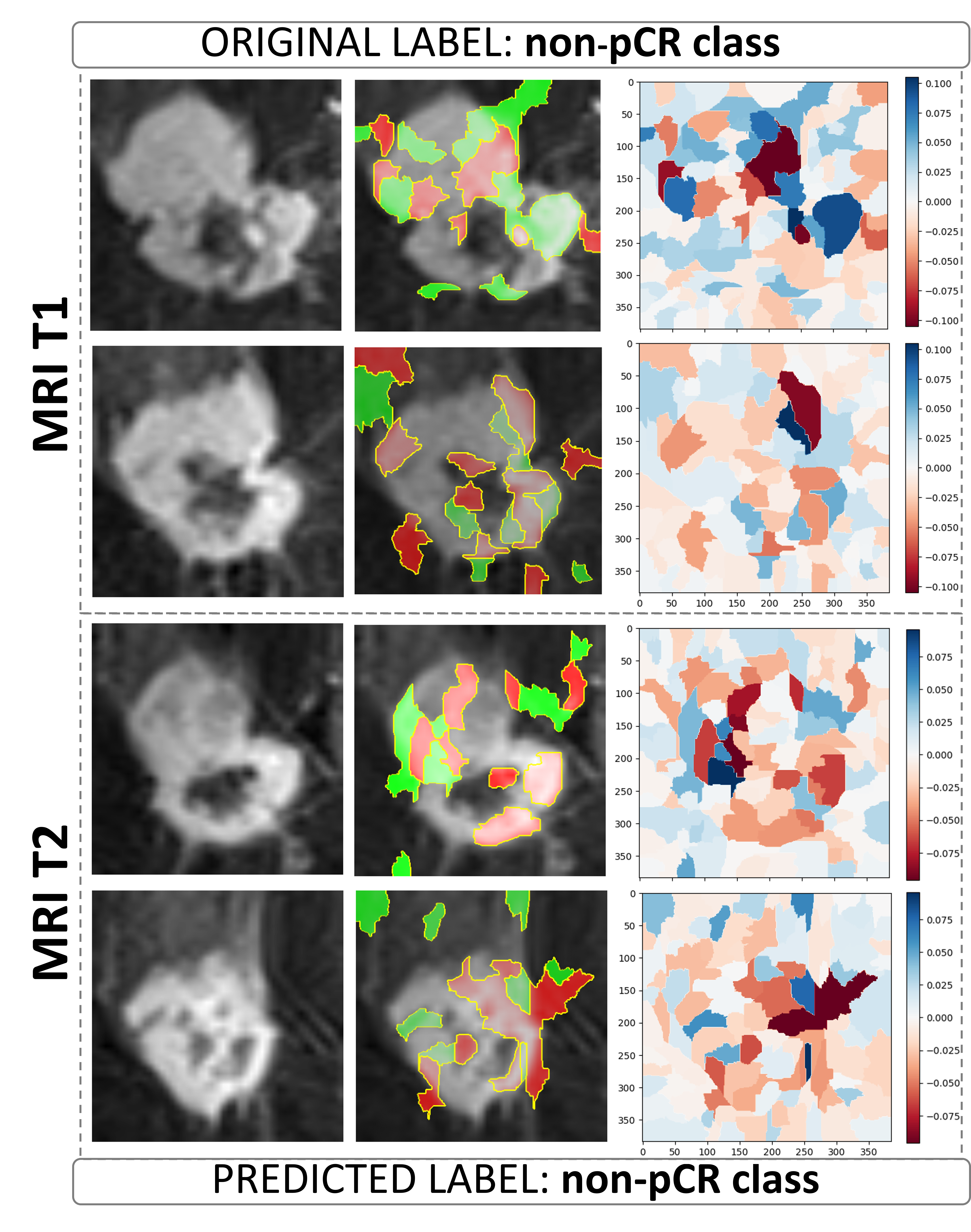

Supplement: Supplementary file 2 — Figure S2. Visualization of LIME superpixels in positive and negative regions applied to two BROI slices from a non‐pCR patient in the independent test, correctly classified by both the MRI T1 and MRI T2 models utilizing a transfer learning module based on the ViT architecture. The panels on the left show the raw slices. The central ones depict the raw slices overlaid by the most contributing superpixels, where the red color highlights the negatively contributing superpixels to the assignment to non‐pCR class, whereas the green represents otherwise. The panels on the right represent heatmaps where color intensity is a measure of importance of all the superpixels generated on the raw slices (blue for a positive contribution, and red for a negative contribution). [file CAM4-13-e70482-s003.tif]

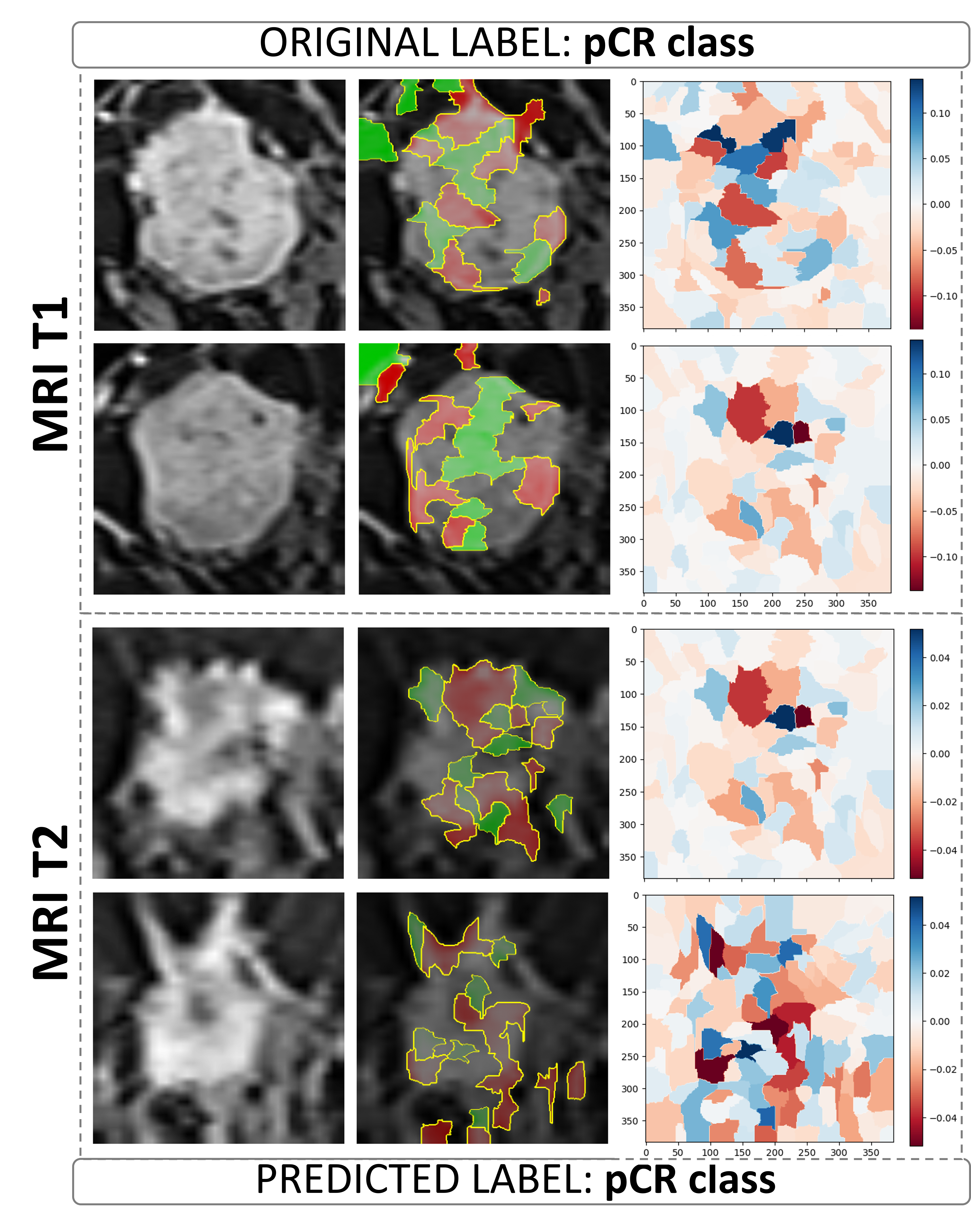

Supplement: Supplementary file 3 — Figure S3. Visualization of LIME superpixels in positive and negative regions applied to two BROI slices from a pCR patient in the independent test, correctly classified by both the MRI T1 and MRI T2 models utilizing a transfer learning module based on the ViT architecture. The panels on the left show the raw slices. The central ones depict the raw slices overlaid by the most contributing superpixels, where the red color highlights the negatively contributing superpixels to the assignment to non‐pCR class, whereas the green represents otherwise. The panels on the right represent heatmaps where color intensity is a measure of importance of all the superpixels generated on the raw slices (blue for a positive contribution, and red for a negative contribution). [file CAM4-13-e70482-s005.tif]

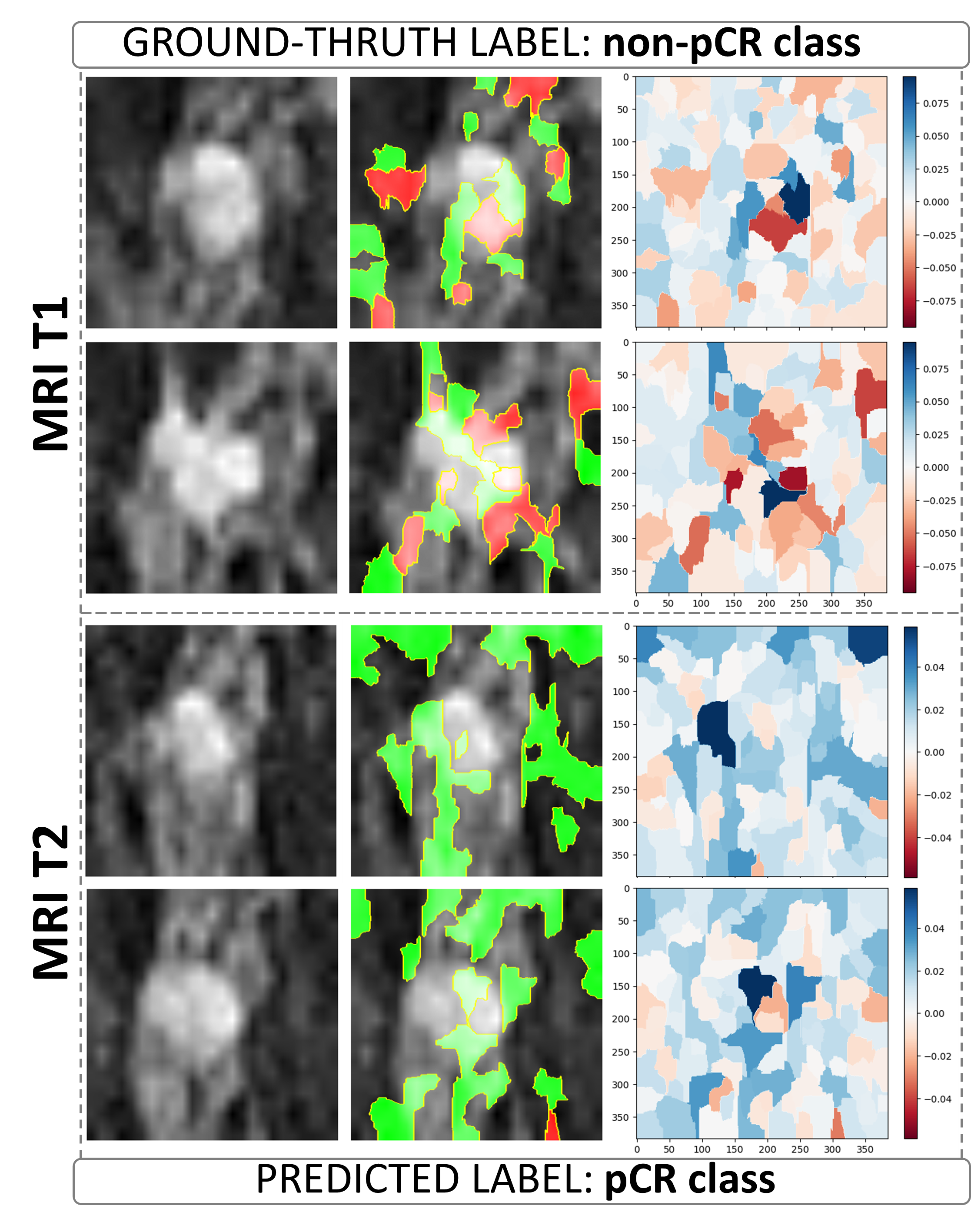

Supplement: Supplementary file 4 — Figure S4. The visualization of LIME superpixels in positive and negative regions applied to two BROI slices related to a non‐pCR patient misclassified by both MRI T1 and MRI T2 models with a transfer learning module as ViT architecture. The left panels show the raw slices. The central panels depict the raw slices overlaid by the most contributing superpixels, where the red color highlights the negatively contributing superpixels to the assignment to non‐pCR class, whereas the green represents otherwise. The right panels represent heatmaps where color intensity is a measure of importance of all the superpixels generated on the raw slices (blue for a positive contribution, and red for a negative contribution). [file CAM4-13-e70482-s004.tif]

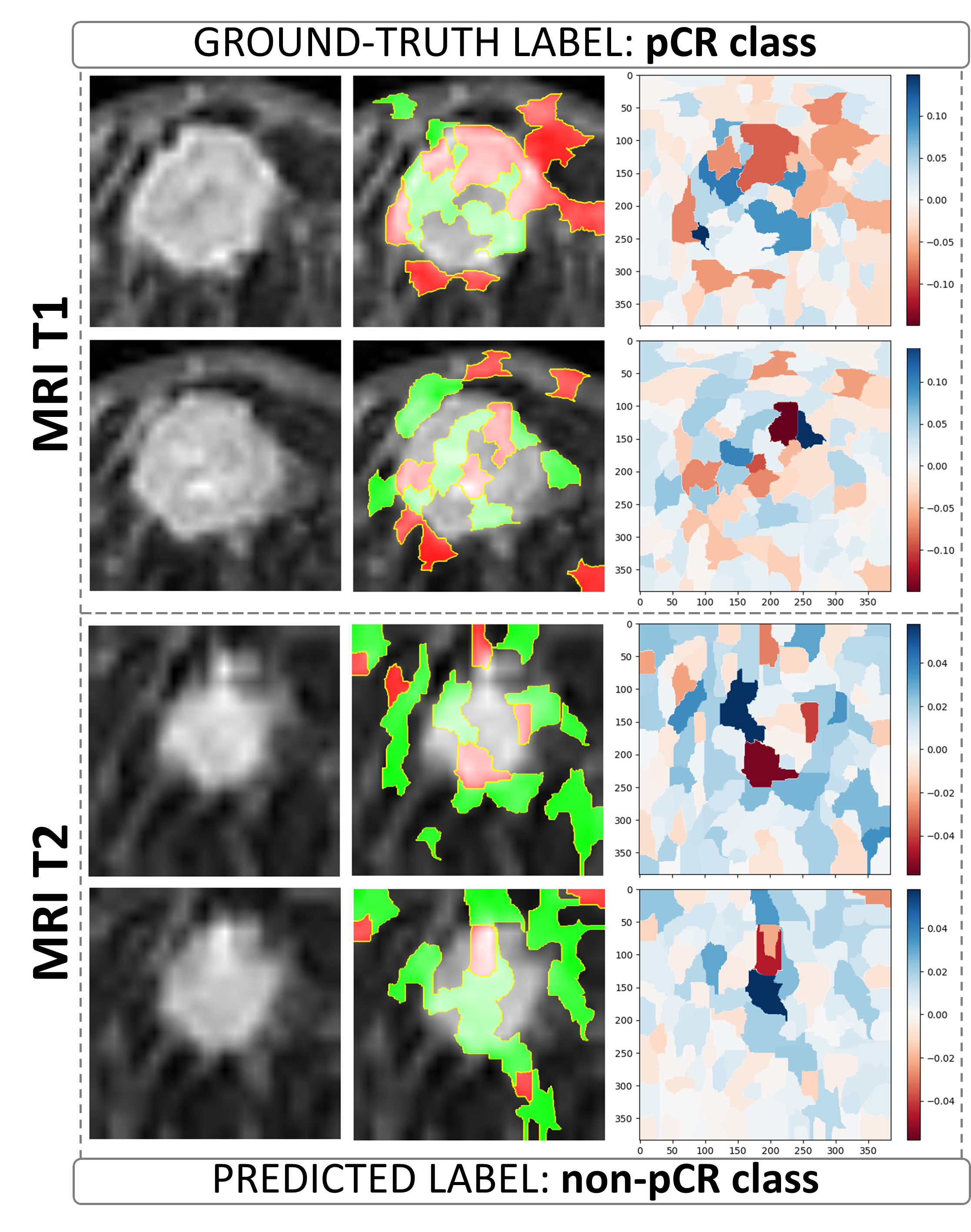

Supplement: Supplementary file 5 — Figure S5. The visualization of LIME superpixels in positive and negative regions applied to two BROI slices related to a pCR patient misclassified by both MRI T1 and MRI T2 models with a transfer learning module as ViT architecture. The left panels show the raw slices. The central panels depict the raw slices overlaid by the most contributing superpixels, where the red color highlights the negatively contributing superpixels to the assignment to non‐pCR class, whereas the green represents otherwise. The right panels represent heatmaps where color intensity is a measure of importance of all the superpixels generated on the raw slices (blue for a positive contribution, and red for a negative contribution). [file CAM4-13-e70482-s001.tif]
